# A persistently infecting coronavirus in hibernating *Myotis lucifugus,* the North American little brown bat

**DOI:** 10.1099/jgv.0.000898

**Published:** 2017-08-25

**Authors:** Sonu Subudhi, Noreen Rapin, Trent K. Bollinger, Janet E. Hill, Michael E. Donaldson, Christina M. Davy, Lisa Warnecke, James M. Turner, Christopher J. Kyle, Craig K. R. Willis, Vikram Misra

**Affiliations:** ^1^​ Department of Veterinary Microbiology, University of Saskatchewan, Saskatoon, Saskatchewan, Canada; ^2^​ Department of Pathology, University of Saskatchewan, Saskatoon, Saskatchewan, Canada; ^3^​ Trent University, Peterborough, Ontario, Canada; ^4^​ Department of Biology, University of Winnipeg, Winnipeg, Manitoba, Canada

**Keywords:** bats, *Myotis lucifugus*, coronavirus, persistent infection

## Abstract

Bats are important reservoir hosts for emerging viruses, including coronaviruses that cause diseases in people. Although there have been several studies on the pathogenesis of coronaviruses in humans and surrogate animals, there is little information on the interactions of these viruses with their natural bat hosts. We detected a coronavirus in the intestines of 53/174 hibernating little brown bats (*Myotis lucifugus*), as well as in the lungs of some of these individuals. Interestingly, the presence of the virus was not accompanied by overt inflammation. Viral RNA amplified from little brown bats in this study appeared to be from two distinct clades. The sequences in clade 1 were very similar to the archived sequence derived from little brown bats and the sequences from clade 2 were more closely related to the archived sequence from big brown bats. This suggests that two closely related coronaviruses may circulate in little brown bats. Sequence variation among coronavirus detected from individual bats suggested that infection occurred prior to hibernation, and that the virus persisted for up to 4 months of hibernation in the laboratory. Based on the sequence of its genome, the coronavirus was placed in the *Alphacoronavirus* genus, along with some human coronaviruses, bat viruses and the porcine epidemic diarrhoea virus. The detection and identification of an apparently persistent coronavirus in a local bat species creates opportunities to understand the dynamics of coronavirus circulation in bat populations.

## Introduction

In recent years, three coronaviruses (CoV) have emerged to have a significant impact on global health and the global economy. Two of these, which cause severe acute respiratory syndrome (SARS [1]) and Middle Eastern respiratory syndrome (MERS [2–5]), are human pathogens. The SARS outbreak in 2002–2003 led to 8096 cases, with 10 % mortality in 27 countries [6]. Since September 2012, about 1900 cases of MERS have been reported, with a mortality rate of about 35 % [7]. The third coronavirus, porcine epidemic diarrhoea coronavirus (PED-CoV [8]), was introduced into North American commercial pig herds and led to an economic loss of almost 2 billion dollars in the United States [9]. These three coronaviruses are believed to have spilled over from bats, because similar coronaviruses have been detected in bats [10, 11]. Interestingly, while coronaviruses cause serious and often fatal disease in their secondary hosts, such coronaviruses do not cause any clinical disease in their putative reservoir hosts, i.e. bats [12–14]. The reasons for this difference in outcomes for coronavirus infection, and the factors that lead to virus spillover, are not clearly understood. There are numerous studies on the pathogenesis of SARS-CoV, MERS-CoV and PED-CoV in humans and pigs [15–18], but there are few reports examining coronavirus interactions with their primary bat hosts [13, 14]. Our goal was, therefore, to identify coronaviruses in a common and widespread North American bat species and study virus–bat interactions within this species.

Access to a large number of archived samples from an unrelated experiment [19–21] gave us the opportunity to screen little brown bat tissues for the presence of coronaviruses, to determine the sequence of the genome of the virus and identify specific tissues for which the virus has a predilection. Our results suggest that about a third of little brown bats are infected with several distinct clades of an *Alphacoronavirus*, and that the bats retain the virus for up to 4 months of hibernation. Since the bats we examined had been maintained as groups in isolated incubators or semi-isolated cages, we were able to test the hypothesis that little brown bats in the wild are infected with closely related variants of a coronavirus. We predicted that, since the bats were randomly assigned to different incubators/cages, (1) prevalence of infection should be similar across incubators and cages, and (2) any variation in viral genomes should be evenly distributed among cages and incubators.

## Results

### Detection of a coronavirus in hibernating little brown bats

To estimate the prevalence of coronavirus in little brown bats, and to determine the tropism of the virus, we performed PCR for the coronavirus RNA-dependent RNA polymerase (RdRp) gene on samples from the brain, intestines, liver, kidney and spleen of 157 little brown bats. We only detected the coronavirus in the intestines. On average, one-third of the bats (Table 1) contained detectable coronavirus RNA. There was no difference in the prevalence of virus between experimental groups in the different cages/incubators (Chi-square test, *n*=174, *P* value=0.817). A lack of blood samples prevented us from confirming viral prevalence by serological methods.

**Table 1.** Prevalence of the *Myotis lucifugus* bat coronavirus (Myl-CoV) in little brown bat intestines, based on the detection of a portion the viral RdRp gene

Year	Incubator name	No. of positive bats/no. of bats tested	Percentage positive
2011	Incubator A	7/18	39
2011	Incubator B	3/13	23
2011	Incubator C	7/16	44
2012	Incubator DC	6/21	29
2012	Incubator DI	9/23	39
2012	Incubator EC	3/10	30
2012	Incubator EI	2/10	20
2012	Incubator FC	4/10	40
2012	Incubator FI	2/11	18
2012	Incubator GC	2/10	20
2012	Incubator GI	4/11	36
2012	Incubator HC	3/11	27
2012	Incubator HI	1/10	10
**Total**		**53/174**	**30**

**Fig. 1.**
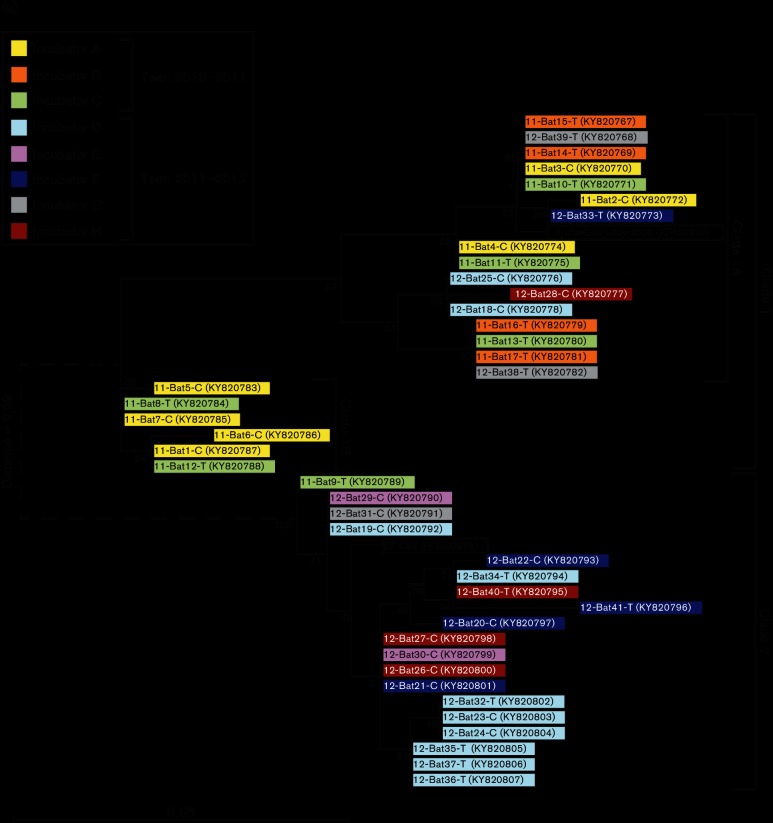

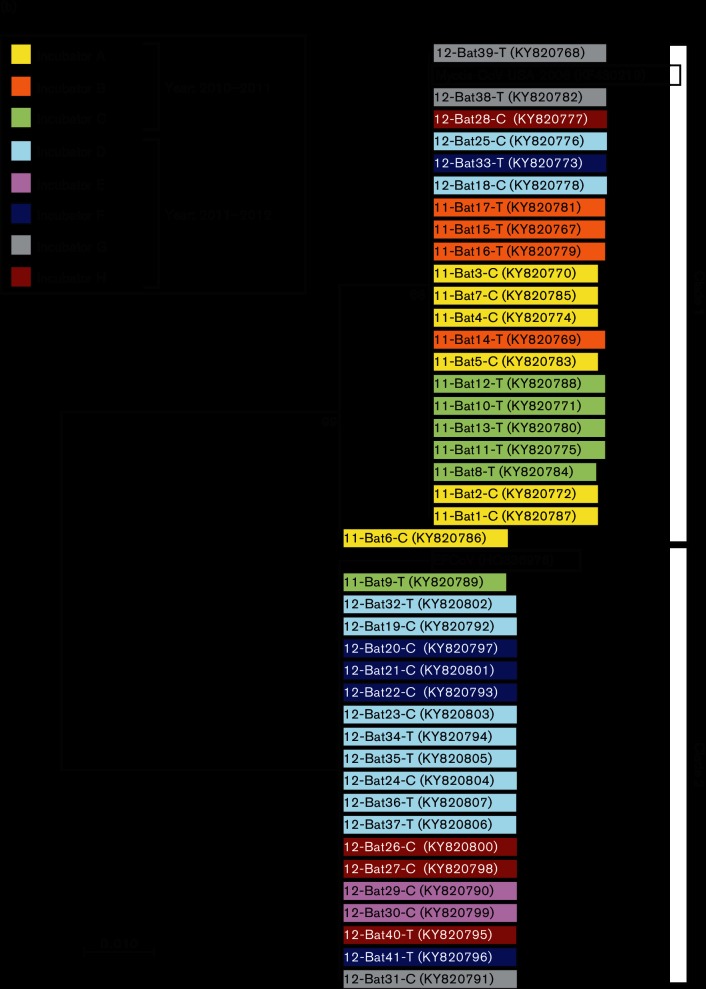

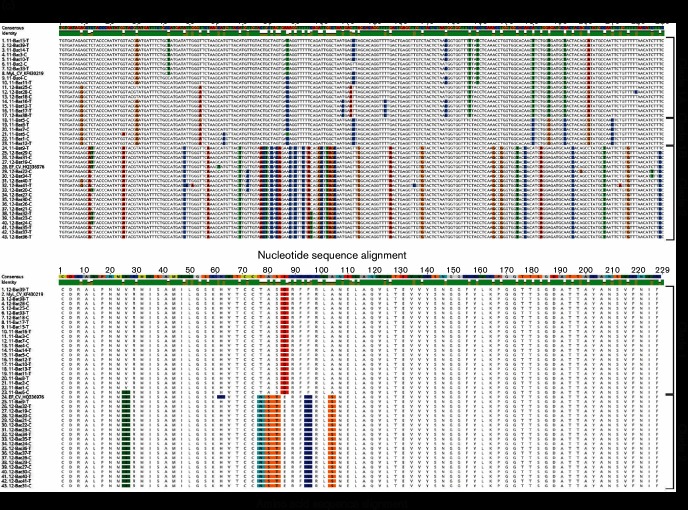
Comparison of nucleotide and amino acid sequence of PCR products from a 229 bp portion of the coronavirus polymerase gene amplified from the intestine of bats. Groups of bats were isolated in incubators with little likelihood of cross-infection during hibernation. (a) Maximum-likelihood tree of nucleotide sequence variation. The colour of the box indicates the bat’s hibernation incubator. The first two digits indicate the year of the experiment (2010–2011 or 2011–2012) and the bat’s identification number. For 2011–2012 each incubator contained two cages, designated either T or C. Corresponding sequences from a coronavirus from *Myotis lucifugus* (Myotis-CoV-USA-2006, GenBank accession number KF430219) and from *Eptesicus fuscus* (HQ336976) were included in the analysis and are in white boxes. The numbers at the nodes are the bootstrap values (per cent) obtained for 1000 replicates. The inter-clade distance (the distance between clades 1 and 2) was much larger than the intra-clade distances (the distance between individual coronavirus sequences within a clade), and therefore it has been depicted with a dotted line and is not to scale. (b) Maximum-likelihood tree for the amino acid sequence derived from the nucleotide sequences analysed in (a). (c) The clustalw alignments used to generate the trees for nucleotide and amino acid sequences. Residues that differ from the consensus are highlighted in colour.

We then compared the nucleotide sequences and the derived amino acid sequences of the PCR products. We included a corresponding segment from little brown bat coronavirus (KF430219) and from the Rocky Mountain coronavirus detected in a big brown bat (HQ336976) into the alignments. The sequences segregated into two distinct clades (Fig. 1a). The clade 1 sequences were most similar to KF430219 and the clade 2 sequences resembled sequences from HQ336976. Most of the sequence differences within each clade were synonymous (Fig. 1b), while there were seven amino acid differences between clades 1 and 2 (Fig. 1c). Except for some bats in incubator D, the nucleotide inter-clade and intra-clade polymorphisms were scattered among the hibernation incubators. This suggested that the bats were infected before being placed in the incubators, rather than acquiring infection from the incubator or cage mates.

### Complete genome sequencing and phylogenetic analysis

We assembled the entire genome of the coronavirus from RNAseq data from the intestines of seven bats (which contained clade 1 coronaviruses). The genome of the *Myotis lucifugus* bat coronavirus (Myl-CoV) is 28 173 bases. We assigned open reading frames based on the published KF430219 sequence. The assembled sequence includes a 3′ poly-adenine tail, which is missing from the annotated KF430219 sequence.

**Fig. 2.**
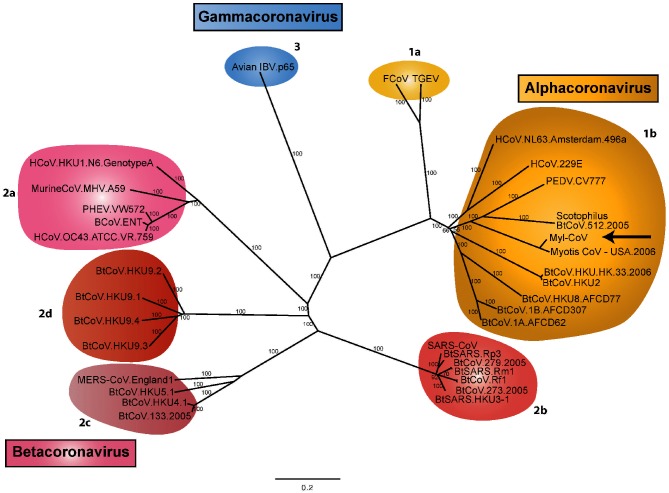
Whole-genome phylogeny comparing Myl-CoV with representative coronaviruses. The whole-genome sequences of 34 coronaviruses, including Myl-CoV, were aligned. Three distinct phylogenetic genera are shown: *Alphacoronaviruses*, *Betacoronaviruses* and *Gammacoronaviruses.* The location of Myl-CoV within *Alphacoronaviruses* is indicated by an arrow. *Deltacoronaviruses* are newly characterized and are not shown. Recognized subgroup clusters are marked as 2a–2d for the *Betacoronaviruses* and 1a and 1b for the *Alphacoronaviruses*.

Phylogenetic analysis of Myl-CoV and other representative coronaviruses places Myl-CoV within the genus *Alphacoronavirus*. Myl-CoV is more closely related to Scotophilus bat coronavirus 512 and PEDV-CoV (CV777 strain). Other *Alphacoronaviruses* related to the Myl-CoV are human CoV 229E, NL63 and coronaviruses detected in other bat species (BtCoV-HKU2,HKU8, HKU-HK33, 1B-AFCD307 and 1A-AFCD62) (Fig. 2).

### Detection of Myl-CoV in bronchial epithelium of bats

**Fig. 3.**
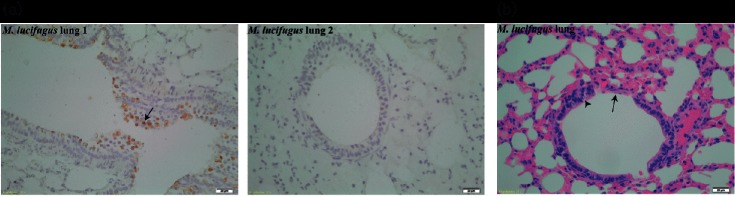
(a) Cells immuno-stained for Myl-CoV nucleocapsid in the bronchial epithelium of lungs. Immunohistochemistry performed using Myl-CoV N antiserum. Lung 1 is from a bat with coronavirus detected in its intestine. Lung 2 is from an uninfected bat. Cells stained for Myl-CoV N protein (indicated by arrows) were vacuolated and in some cases sloughed into the bronchial lumen. Only bronchial epithelial cells had detectable viral antigen. (b) Pathology of Myl-CoV-infected lung. H and E staining of bat lung infected with coronavirus. Vacuolation of infected cells (shown by an arrowhead) was present along the bronchial epithelium. A portion of bronchial epithelium was sloughed off (shown by an arrow). Neutrophils were observed in the vicinity of the infected portion of the bronchus, but the overall inflammation in the entire lung was low.

Several coronaviruses have a predilection for respiratory as well as intestinal tissue. Although we had only detected Myl-CoV RNA in intestinal samples, we performed immunohistochemistry (IHC) to further explore the tropism of the virus. We were unable to detect coronavirus antigen in any of the tissues except for lungs. We detected the Myl-CoV antigen in the lung of five bats, all of which were positive for viral RNA in the intestines (Fig. 3a). In the lungs, the Myl-CoV antigen was only present in the bronchial epithelial cells. Cells containing nucleocapsid antigen showed degenerative changes in the form of vacuolation, and some cells appeared to have exfoliated.

**Fig. 4.**
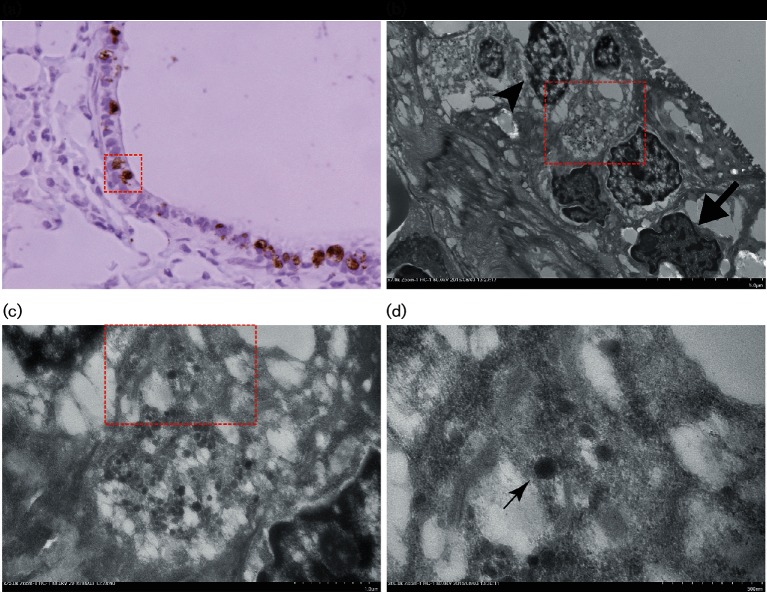
Transmission electron micrograph showing coronavirus-like particles in the bronchial epithelial cells. (a) A haematoxylin and immune-stained (Myl-CoV N protein) section of lung. The red square denotes the portion picked from a consecutive H and E section for electron microscopy. (b) Electron micrograph of cells selected in (a). Successive enlargement of portions of the section in a box with red dotted lines (c), d). Particles (around 125 nm in size) were observed in the bronchial epithelium (thin arrow). Those cells which contained the virus-like particles showed nuclear degradation (indicated by the arrowhead). The uninfected cell nucleus shows normal morphology (shown by thick arrow).

We also performed transmission electron microscopy on portions of the lung sections that contained viral antigen (Fig. 4). Although the quality was compromised due to formalin fixation, we observed electron-dense particles in the cells that were positive for Myl-CoV antigen. The size of these particles was as expected for coronaviruses (approximately 125 nm [22]). Cellular degenerative changes were evident in the cells containing the particles. In the same section, other cells were healthy and did not contain such particles. Furthermore, the presence of viral RNA was confirmed by performing PCR on lung cDNA (obtained from RNA) using the primers against Myl-CoV nucleocapsid gene. Sanger sequencing of the amplified PCR product showed a match with Myl-CoV nucleocapsid sequence.

### Pathology in bat lung due to Myl-CoV infection

To assess the pathology due to coronavirus infection, histological sections of the Myl-CoV-positive lungs of *M. lucifugus* were evaluated independently by two pathologists. Although the lesions were very mild, both pathologists had higher lesion scores for the categories of bronchiolar epithelial vacuolation and degeneration, bronchiolar epithelial hyperplasia and erosion of epithelium in virus-infected bats compared to uninfected bats. There was no obvious consistent inflammation of the bronchus and, although there was mild diffuse inflammation of the interstitium and alveoli, there was no difference between virus-infected and uninfected bats (Fig. 3b).

## Discussion

Although events of successful viral spillover to distantly related species are thought to be extremely rare, in recent years coronaviruses, including SARS-CoV [1], MERS-CoV [2–5] and PED-CoV [8], have spilled over from bats to other species. Circumstantial evidence suggests that most alpha and beta coronaviruses parasitizing other mammals may also have originated in bats [23]. Little is known at present about the dynamics of coronavirus infection in the reservoir bat hosts, or how the viruses are spread from bat to bat, or from bats to other mammals. In this study, we examined a coronavirus in its natural host, the little brown bat. This study system provides a useful model for understanding factors that may promote spillovers. Our results suggest the following: (a) the *Myotis* coronavirus (Myl-CoV) is mainly present in the intestines and lungs; (b) co-hibernating little and big brown bats may share closely related coronaviruses; (c) Myl-CoV can persist in *M. lucifugus* for up to 4 months, the hibernation period of our experiment; and (d) the presence of Myl-CoV in bronchial epithelium is associated with minimal pathology or inflammation. However, a larger sample size under controlled conditions of exposure, with more rapid fixation of tissues, is required to fully characterize the lesions in lung and intestines.

Dominguez *et al.* [24] and others [20] previously detected a coronavirus in little and big brown bats, suggesting that the virus may infect bats of both species, crossing between the bats at shared co-hibernation sites. The virus originally detected in a big brown bat was named the Rocky Mountain coronavirus [24] for the location of its initial detection. The RNA amplified from little brown bats in this study appeared to be from two distinct clades. The sequences in clade 1 were very similar to the archived sequence derived from little brown bats and the sequences from clade 2 were more closely related to the archived sequence from big brown bats. This suggests that two closely related coronaviruses may circulate in little brown bats. Although the sequence traces did not suggest a mixture of PCR products, we cannot completely rule out the possibility that the individual bats were infected with viruses from both clades, and that the PCR likely favoured the predominant viral component.

Complex strategies allow viruses to remain endemic in populations. These include: (1) a continuous source of susceptible hosts for viruses that cause short-term acute infections with long-lasting immunity (e.g. measles virus); (2) antigenic drift (e.g. influenza virus) of virus or waning immunity (e.g. respiratory syncytial virus) that allows reinfection; and (3) long-lasting latent (e.g. herpesviruses) or persistent infections (e.g. pestiviruses) with sustained or periodic shedding. How bat viruses are maintained in their natural host populations, or how they avoid extinction as host populations become immune and less susceptible, however, is not understood. It is possible to establish persistent infections in cultured cells with viruses that may have originated in bats, including Ebola virus [25] and SARS-CoV [26, 27], but whether these viruses persist in their primary hosts is not known. The results of studies aimed at determining whether bat viruses persist in infected bats are controversial. There is no direct evidence for either persistence or transmission dynamics, and this is a knowledge gap in bat-virus ecology that needs to be addressed [28].

Based on the sequence of the amplified portion on the RdRp gene, we observed considerable polymorphism among the Myl-CoV, with sequences segregating into distinct clades. Based on the maximum mutation rate possible for the RdRp gene [29], we should only have observed 1.16 random mutations in the 229 bp segment. Most of the intra- and inter-clade polymorphisms exceeded this rate. Also, while the viruses detected in 2011 were primarily clade 1 and the 2012 viruses were primarily clade 2, both clades were detected in samples from either year. The differences between clades likely represent fixed nucleotide polymorphisms rather than random changes. Although recombination of coronavirus clades is possible, performing deep sequencing on the samples would enable us to negate this possibility. Ge and others also found the co-existence of a variety of coronavirus in bat colonies [30].

Osborne and others [23] were unable to detect virus in the rectal swabs of individual bats sampled over time in an extensive survey of New World *Alphacoronaviruses*. They concluded that these coronaviruses do not persist in their hosts, but are maintained in populations by the introduction of new susceptible individuals. Their results, however, do not rule out persistence in individual animals with low levels of virus replication and undetectable shedding, interspersed with short periods of increased replication and shedding. Our observations suggest that the Myl-CoV can persist in its hosts for at least the 4-month hibernation period. Due to strict biosecurity, spread of virus between incubators was unlikely and the distribution of variants among incubators (and cages) argues against spread within incubators (or cages). Our results therefore suggest that the bats were infected before they were collected.

Whole-genome phylogenetic analysis revealed that the Myl-CoV belongs to the genus *Alphacoronavirus,* which includes three coronaviruses that infect human lungs and pig intestines (HCoV-229E, HCoV-NL63 and PED-CoV). We confirmed the presence of virus in the intestine via PCR and in the lungs by immunohistochemistry, electron microscopy and PCR. However, we were unable to detect the Myl-CoV in the intestines using immunohistochemistry. The reason for this might be that the part of the intestine used for RNA extraction had the virus in it, whereas the part used for histology did not.

Our histological lung sections provided a novel insight into the persistent infection of a coronavirus in its reservoir bat host. Firstly, we observed that the cells that were infected showed degenerative changes that resulted in rare multifocal areas of bronchiolar epithelial erosions, with no obvious inflammation targeting these lesions. The absence of neutrophilic infiltration is contrary to what has been seen in non-bat species affected by similar coronaviruses. Previous studies in transgenic mice showed that HCoV-229E infection led to massive neutrophilic infiltrate [31]. Lung samples from piglets infected with PED-CoV showed the presence of moderate neutrophilic infiltrate (even though PED-CoV has a predilection for intestines) [32]. Hibernating bats do appear to be capable of a local inflammatory response following fungal infection [33]. A low level of neutrophilic infiltration in coronavirus-infected lungs reinforces the fact that bats are unique in the way that they respond to a coronavirus infection. Lower inflammation might be due to fewer chemotactic factors being produced as a result of infection, which might be an inherent feature of *M. lucifugus*. It might also be that bat neutrophils are more efficient at controlling virus infection and obviating a massive neutrophilic infiltrate. An alternative explanation for the lack of inflammation in bats may be that cell necrosis occurs at the epithelial surface, with dead cells sloughing into the lumen. The cytokines required to stimulate inflammation and immunity might not enter the interstitium and the systemic circulation. An infection that is localized to epithelial cells without breaching the basement membrane is a good strategy for a virus to allow persistence of infection. However, in our study the sample size was small and it is difficult to reach a firm conclusion about the host’s response to coronavirus infection.

**Fig. 5.**
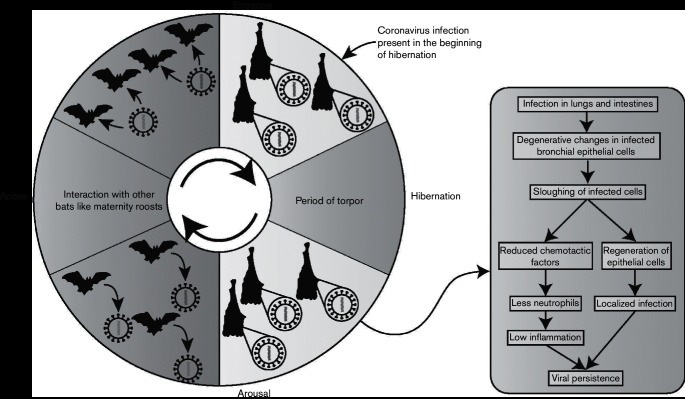
Proposed model of coronavirus maintenance in bat populations. Bats are infected with one of the closely related coronaviruses that are distinguishable from each other by minor nucleotide polymorphisms. The virus then persists at low levels during hibernation. Due to limited replication in bronchial epithelial cells, there is little host response against the virus, favouring persistence. Naïve and susceptible young individuals acquire the virus in maternity roosts when viral replication and shedding increases, caused by hormonal changes or other stress factors Bats enter hibernation with the infection present in them. During hibernation, the virus persists due to low levels of physiological and physical activity, and low levels of inflammation.

Although our results demonstrate viral persistence during hibernation and do not address it in normothermic bats, we propose a model for the maintenance of the coronavirus in little brown bat populations (Fig. 5). Bats are infected with one of the closely related coronaviruses that are distinguishable from each other by minor nucleotide polymorphisms. The virus then persists at low levels, probably undetectable in faecal material. Due to limited replication in bronchial epithelial cells, there is little host response against the virus, favouring persistence. Naïve and susceptible young individuals acquire the virus in maternity roosts when viral replication and shedding increases, caused by hormonal changes or other stress factors [33, 34].

Our study demonstrates for the first time that several bats in a population can maintain a coronavirus infection through hibernation as an apparently non-pathogenic infection. Our observations also support growing evidence that natural and experimental viral infections in bats are not accompanied by acute inflammation and pathology.

## Methods

### Bats

Bat tissues were obtained from two previous experiments [19, 21] in the winters of 2010–2011 and 2011–2012, designed to study the pathogenesis of the fungus, *Pseudogymnoascus destructans*, the causal agent of WNS. Male little brown bats were collected from two different WNS-free caves in Manitoba, Canada under the Manitoba wildlife scientific permits WB11145 and WB13148. Details for the segregation of bats into incubators and cages in the 2010–11 and 2011–12 experiments are in Table 2. Bats were euthanized at the end of the experiment i.e. after 4 months (2010–11) and 3 months (2011–12). Bats that succumbed to the WNS fungus infection were removed prior to the end of the experiment. Immediately following euthanasia, samples from the brain, intestines, liver, kidney and spleen were preserved in RNAlater (Qiagen, cat #76106) or in formalin [21].

**Table 2.** Segregation of hibernating bats

Year	Incubator name and cage Name (if applicable)	Number of bats	Inoculation type	Incubator status
2010–2011	A	18	Control	Relative humidity >97 %; temperature 7 °C
	B	18	Fungus-infected (European strain)	Relative humidity >97 %; temperature 7 °C
	C	18	Fungus-infected (American strain)	Relative humidity >97 %; temperature 7 °C
2011–2012	D (C)	21	Control	Relative humidity >97 %; temperature 7 °C
	D (I)	23	Fungus-infected	
	E (C)	10	Control	Relative humidity 99 %; temperature 7 °C
	E (I)	10	Fungus-infected	
	F (C)	10	Control	Relative humidity 95 %; temperature 7 °C
	F (I)	11	Fungus-infected	
	G (C)	10	Control	Relative humidity 90 %; temperature 7 °C
	G (I)	11	Fungus-infected	
	H (C)	11	Control	Relative humidity 85 %; temperature 7 °C
	H (I)	10	Fungus-infected	

### RNA extraction and cDNA preparation

Bat tissues were homogenized and RNA was extracted using the RNeasy Plus mini kit (Qiagen, cat #74136), as per the manufacturer’s protocol. cDNA was prepared using the QuantiTect reverse transcriptase kit (Qiagen, cat #205313) as per the protocol mentioned in the kit.

### Polymerase chain reactions (PCRs) and plasmid constructions

The sequences for all of the primers used in the PCRs are in Table 3. Semi-nested PCR primers amplified a portion of the RdRp region of the coronavirus genome [20] from cDNA prepared from bat tissues. For the primary and secondary reactions, the expected products were 441 and 273 bp, respectively. All of the amplified products were sequenced using the amplification primers. These sequences have been submitted to GenBank, accession numbers KY820767 to KY820807.

**Table 3.** Primers used for PCR

Forward primer	Reverse primer	Purpose	Elongation time	Product length
5′-CCATCATCAGATAGAATCATC-3′	5′-TGGTTGGGACTATCCTAAGTG-3′	Primary reaction for coronavirus detection	1 min	441 bp
5′-CGGTTCACATTAGCACTGACAG-3′	5′-TGGTTGGGACTATCCTAAGTG-3′	Secondary reaction for coronavirus detection	1 min	273 bp
5′-ATG GCC TCT GTT AAG TTC GCC AA-3′	5′-TTAAGCTGTGCTCTGAGAATT-3′	Coronavirus nucleocapsid amplification for TOPO-TA cloning	1 min 30 s	1278 bp
5′-GCCGGATCCATGGCCTCTGTTAAGTTCGCCAA-3′	5′-GCCTCTAGATTAAGCTGTGCTCTGAGA-3′	Coronavirus nucleocapsid cloning into pGEX-KG vector	1 min 30 s	1296 bp
5′-GCCAAGCTTATGGCCTCTGTTAAGTTC-3′	5′-GCCTCTAGATTAAGCTGTGCTCTGAGA-3′	Coronavirus nucleocapsid cloning into P3X-FLAG vector	1 min 30 s	1296 bp

The *Myotis lucifugus* coronavirus (Myl-CoV) nucleocapsid gene (1278 bp) was amplified from intestines and lungs using a forward primer and a reverse primer (Table 3). The veracity of the PCR products was confirmed by sequencing and the products were cloned into pCR 2.1-TOPO vector using the TOPO TA cloning kit (Invitrogen, cat #450641). The sequences were also cloned into the pGEX-KG protein expression plasmid [a gift from Gerry Weinmaster (University of California, Los Angeles, CA, USA)] using the *Bam*HI and *Xba*I restriction sites at 5′ and 3′ ends, respectively.

### High-throughput sequencing and assembling the Myl-CoV genome

Total RNA from seven bats (four from incubator A and three from incubator B) was sent to the Centre for Applied Genomics at the Hospital for Sick Children (Toronto, Canada), where RNA quality was assessed using a Bioanalyzer (Agilent Technologies), Poly(A) mRNA was enriched using oligo dT-beads, and cDNA libraries were prepared using the NEBNext ultra directional RNA library prep lit (New England Biolabs). Barcoded libraries were pooled in equimolar quantities and sequenced on a HiSeq 2500 system (Illumina) to generate 126 bp paired-end reads.

Sequence data quality was assessed using FastQC v 0.11.5 [35] and the reads were trimmed to remove adapter sequences and low-quality bases using Trimmomatic v 0.36 [36] using the following settings: Illumina clip: 2 : 30 : 10, leading : 3, tailing : 3, slidingwindow : 4 : 15 and minlength : 36. To identify the host sequences, we used TopHat v 2.1.1 [37] to align trimmed reads to the Ensembl *M. lucifugus* genome sequence assembly (Myoluc 2.0) [38] in the strand-specific mode (fr-firststrand). We sorted the unmapped bam files using samtools v 1.2 [39], extracted the non-host sequences using bedtools v 2.26.0 bamtofastq [40], and pooled the resulting sequences for transcript fragment (transfrag) assembly. We used Trinity v 2.2.0 to generate transfrags using the default parameters and *in silico* read normalization. We performed local blastn to search for sequence similarity between the Trinity-based transfrags and a coronavirus reference genome (KF430219). The sequence of the entire genome of the Myl-CoV was submitted to GenBank, accession number KY799179.

### Myl-CoV nucleocapsid (N) protein purification

Myl-CoV N-pGEX-KG plasmid was transformed into *Escherichia coli* BL21-competent cells [41]. Five hundred ml of cell culture at OD600=0.84 was induced to express Myl-CoV-N-GST with 1 mM isopropylthio-b-D-galactoside (IPTG) at 28.5 °C for 7.5 h. During protein extraction, the temperature was maintained at +4 °C. BL21 cells were centrifuged down and resuspended using 1 % Triton X-100 in TNE buffer. Ten mg ml^−1^ of lysozyme was added for 15 min to accentuate the process of bacterial cell wall breakdown. One ml of Halt protease and phosphate inhibitor cocktail (Thermo Scientific, cat. #78440) and EDTA was added and the blob of bacteria was sonicated for 60 s on ice. The supernatant was removed after centrifuging the bacteria at 15 000 r.p.m. in a Thermo F18 12×50 rotor (32 000 ***g***) for 20 min and the pellet was treated with 1.5 % N-lauroylsarcosine (Sigma L-9150) to further disrupt the cells and then added onto the supernatant. From later SDS-PAGE analysis, we learned that the N-lauroylsarcosine treatment of the pellet led to the release of fusion protein from the cell. Fusion protein was purified from the supernatant using glutathione–Sepharose 4B beads (GE Healthcare), followed by elution using 10 mM glutathione for 16 h. Elution was performed twice to obtain protein concentrations of 6.1 and 4.3 mg ml^−1^. The protein was verified using SDS-PAGE (Fig. S1a, available with the online Supplementary Material).

### Generation of polyclonal antibodies

Polyclonal Myl-CoV N anti-serum was generated in rabbits. This was carried out in strict compliance with the Canadian Council on Animal Care guidelines (protocol 20090050). Two rabbits were procured by the Animal Care Unit at the Western College of Veterinary Medicine. On days 1, 28, 42 and 56, nucleocapsid protein mixed with Titermax was injected into rabbits. Rabbits were bled to obtain serum 1 day prior to each antigen injection. Binding of antibody was verified using Western blot and immunofluorescence (Fig. S1b, c), after which we proceeded with immunohistochemistry.

### Immunohistochemistry (IHC)

Formalin-fixed paraffin-embedded (FFPE) blocks of *M. lucifugus* tissues were obtained from our previous study [21] and sections (4–5 microns) were cut and mounted onto slides. Tissue sections were incubated twice in xylene for 15 min each and then rehydrated in graded alcohol. Tissue sections were then incubated overnight in 0.5 mM PBS for better antigen retrieval. We added 0.5 % hydrogen peroxide in methanol to tissue sections for 20 min at room temperature to block endogenous peroxidase. After distilled water washing, 500 µl of proteinase K (20 µg in 1 ml) treatment for 20 min at 37 °C was performed to enhance antigen retrieval. Slides were blocked using 1 % bovine serum albumin for 30 min. The serum of the rabbits (56th day bleed) containing anti-Myl-CoV N were used as the primary antibodies for staining virus infected cells (1 : 100 dilution). For every slide processed, we also stained slides with the serum extracted prior to antigen injection (pre-bleed) as a negative control. Formalin-fixed Myl-CoV-N-transfected Efk cells were used as a positive control. Tissue sections were incubated with the primary antibodies for 3 h at room temperature, followed by 3×5 min washes with 0.5 mM PBS. Anti-rabbit IgG conjugated with horseradish peroxidase (HRP) (Zymed) was used as a secondary antibody (1 : 500 dilution) for 30 min at room temperature. After 3×5 min washes with 0.5 mM PBS, colour was developed using 500 µl of diaminobenzidine (30 ul diaminobenzidine, 9 µl 0.5 % hydrogen peroxide and 3 ml 0.5 mM PBS) for 10 min at room temperature. Counterstaining was performed using hematoxylin for 30 s and then decolourized using acetic acid–acetone. Slides were dehydrated using graded alcohol and then treated with xylene before coverslips were applied. Formalin-fixed Myl-CoV-N-transfected *Eptesicus fuscus* kidney (Efk) cells [42] were used as a positive control. Counterstaining was performed using hematoxylin.

### Hematoxylin and eosin (H and E) staining

Tissue sections for histopathology were stained with H and E by Prairie Diagnostic Services at the University of Saskatchewan. Each lung section was independently assessed for bronchiolar epithelial degeneration and inflammation by two veterinary pathologists, blinded to sample identity. Five categories of lesions were established: diffuse lung inflammation, inflammation of bronchus, bronchiolar epithelial vacuolation, bronchiolar epithelial hyperplasia and bronchiolar epithelial erosion. All lung sections from all bats were examined to determine the range of changes and then changes were scored between 0 and 3, with 0 indicating normal and 3 indicating the most severe change within the eight bats examined. The maximum score of 3 would still be considered mild within the typical pathology scoring system describing the severity of inflammatory response: mild, moderate or severe.

### Electron microscopy (EM)

Myl-CoV-infected cells in the bronchial epithelium were marked in the corresponding IHC slides, so that the specific location could be sampled for electron microscopy (EM). The slide was soaked in xylene to remove the coverslip, and soaked in xylene:propylene-oxide (PO) (2 : 1) and (1 : 1) for 30 min and 15 min, respectively, with this being followed by 1 h soaking in PO. PO : Epon (1 : 1) was consistently dropped on the slides for 1 to 2 h followed by pure Epon for 1 h. Labelled capsules were filled and inverted on the tissues on the slide. Polymerization was performed at 65 °C for 24 h. The block was broken off the slide, which lifted the section along with it, and was then sectioned on to 200 mesh copper grids, which were viewed by transmission electron microscope.

### Phylogenetic analysis and sequence alignments

For the whole-genome phylogenetic tree, genome sequences of 33 representative coronaviruses and Myl-CoV were aligned using ClustalW (v 1.83) [43]. Maximum-likelihood trees were constructed with mega7 [44]. Divergence was estimated by Kimura’s two-parameter method. Bootstrapping with 1000 replicates was used to estimate the confidence of the tree nodes. The generated tree was then annotated using Adobe Illustrator CC 2015.

For the phylogenetic tree of the RdRp gene segment, The sequences obtained from Sanger sequencing were used, along with corresponding segments from KF430219 and HQ336976. Only sequences that were completely unambiguous were used in the analysis. The maximum-likelihood trees were constructed in a similar manner to that used for the entire-genome phylogenetic tree.

### Accession numbers

Forty-one sequences of the RdRp gene, using Sanger sequencing, were submitted to GenBank (accession numbers KY820767 to KY820807). The Myl-CoV complete-genome sequence was submitted to GenBank (accession number KY799179). Nucleotide sequences for the phylogenetic tree were obtained from GenBank. The accession numbers of the sequences are AF353511 (PEDV.CV777), AF391541 (BCoV.ENT), AY585228 (HCoV.OC43.ATCC.VR.759), DQ001339 (Avian IBV.p65), DQ011855 (PHEV.VW572), DQ022305 (BtSARS.HKU3-1), DQ071615 (BtSARS.Rp3), DQ412042 (BtCoV.Rf1), DQ412043 (BtSARS.Rm1), DQ415904 (HCoV.HKU1.N6.GenotypeA), DQ445912 (HCoV.NL63.Amsterdam.496a), DQ648856 (BtCoV.273.2005), DQ648857 (BtCoV.279.2005), DQ811789 (TGEV), DQ848678 (FCOV), EF065505 (BtCoV.HKU4.1), EF065509 (BtCoV.HKU5.1), EF065513 (BtCoV.HKU9.1), EF065514 (BtCoV.HKU9.2), EF065515 (BtCoV.HKU9.3), EF065516 (BtCoV.HKU9.4), EF203067 (BtCoV.HKU.HK.33.2006), EU420137 (BtCoV.1B.AFCD307), EU420138 (BtCoV.1A.AFCD62), EU420139 (BtCoV.HKU8.AFCD77), FJ647225 (MurineCoV.MHV.A59), KC164505 (MERS-CoV.England1), NC002645 (HCoV.229E), NC004718 (SARS-CoV), NC009019 (BtCoV.HKU4.1), NC009657 (Scotophilus BtCoV.512.2005), NC009988 (BtCoV.HKU2) and KF430219 (Myotis CoV – USA.2006).

## Supplementary Data

2642 Supplementary File 1
