# Supplementary material for: A persistently infecting coronavirus in hibernating Myotis lucifugus, the North American little brown bat
Source: J Gen Virol. 2017 Aug 25;98(9):2297–309. doi: 10.1099/jgv.0.000898 (PMC7079692; doi:10.1099/jgv.0.000898)
Supplement: Supplementary file 1 [file jgv-98-2297-s001.pdf]

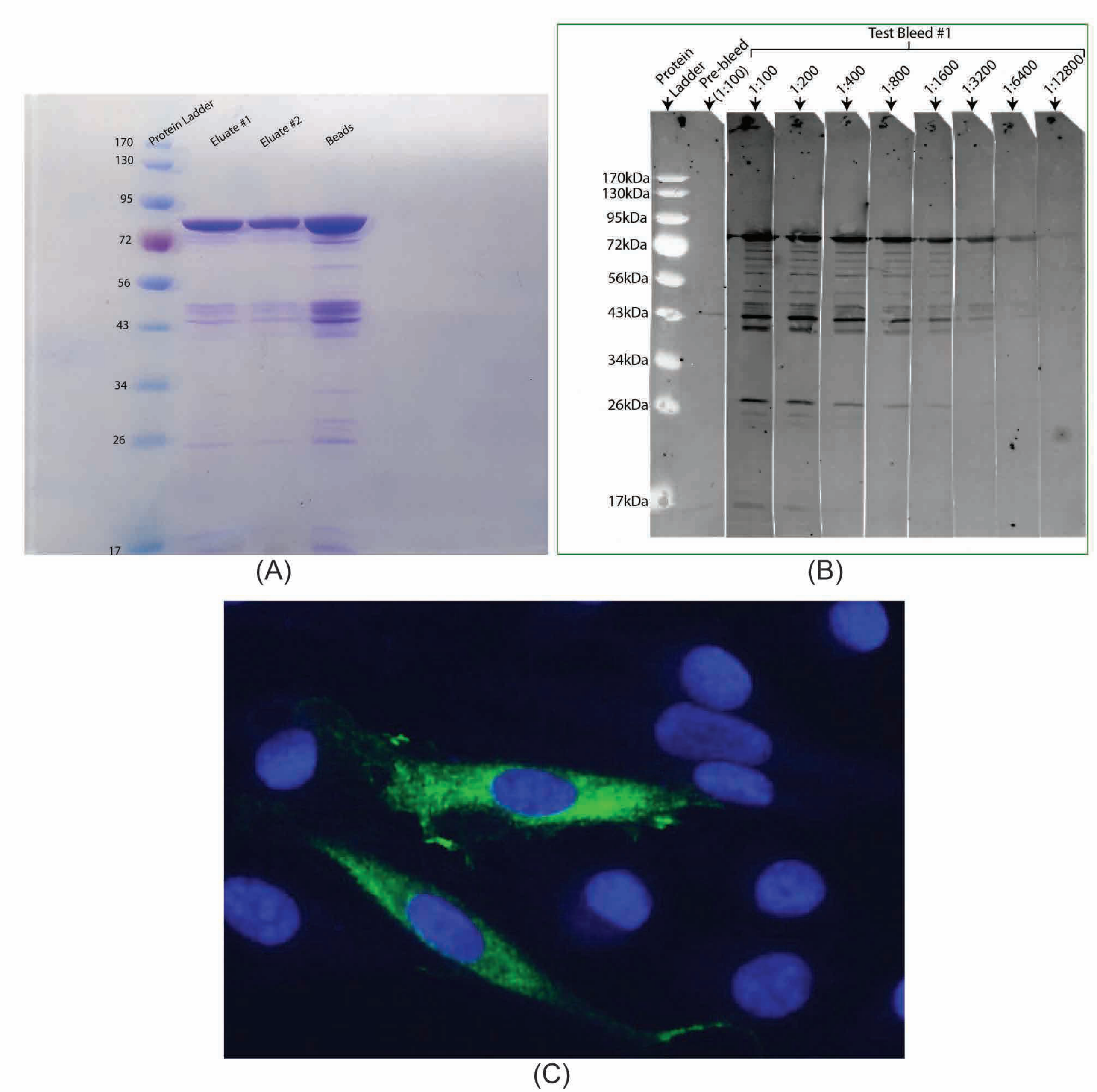

Supplementary Fig. 1. Figure showing the process of developing antibodies. (A) SDS-PAGE showing the fusion protein (Myl-CoV nucleocapsid protein-GST) purified using Glutathione Sepharose 4B beads. Lane 1 contains protein ladder; Lane 2 contains the Eluate#1 (blue band showing the fusion protein); Lane 3 contains the Eluate#2; Lane 4 contains the Beads. (B) Western Blot done using 10ng of Myl-CoV N-GST protein to detect the titers of the antibody in rabbit serum. The first test bleed showed a titer of 6400. (C) Bat cells expressing Myl-CoV N with flag tag stained using rabbit serum (containing Myl-CoV N antibodies). Immunofluorescence assay showing the binding of the antibodies (N protein shown in green).
